# 

**Published:** 1895-10

**Authors:** 


A CONGRESS of Hygiene will be held at Bordeaux, France, in November on the occasion of the exhibition to be held there.

The Ohio College of Dental Surgery has been removed from its old historic locality on College St., Cincinnati, to the north-east corner of Central Avenue and Court St. The old building has been inadequate for the proper accommodation of the College for several years; indeed it has hardly ever been what it ought to be, in regard to light and commodious arrangement. Its surroundings have never been quite satisfactory.

The new location is a very good one indeed, with ample room for any probable requirement; two or three hundred students can be well accommodated; two stories are devoted to the work of the College and these are very well arrainged for all parts of the work. The rooms are light and airy and abundantly large—The building is so located that its light cannot be cut off nor encroached upon.

The change is an excellent one and will certainly add to the facility of the work, making it easier, and consequently better, and more satisfactory to all concerned.

The Faculty is to be congratulated on this change.

### CONTENTS.

### COMMUNICATIONS.

### THE DENTAL REGISTER,

**A MONTHLY MAGAZINE DEVOTED TO THE INTERESTS OF THE**

### DENTAL PROFESSION.

*Terms Reduced to $2.00 Per Year. Single Copies, 25 Cents.*

**EDITED BY PROF. J. TAFT, D.D.S.**

—AND—

**Published by SAM'L A. CROCKER & CO., Ohio Dental and Surgical Depot.**

The volume commences in January and closes in December.

The REGISTER being issued monthly is always fresh, therefore Indispensable to the practitioner who desires to keep posted up with the new inventions and improvements which are daily developed. Neither expense nor effort will be spared to give value and variety to its contents. Articles will be illustrated with well executed engravings whenever they will add to the interest or assist to explanation.

Its extensive circulation and frequency of issue make it one of the BEST DENTAL ADVERTISING MEDIUMS in the United States.

All subscriptions must begin with January or July.

Local or special notices, five lines or less, will be inserted for $3 each insertion; over five lines, 50 cts. per line.

All advertising bills payable in advance, or at furthest, after the issue of the first number containing the advertisement. All transient advertisements to be paid for in advance.

☞CONTRIBUTIONS SOLICITED.

Exclusively Editorial Communications should be addressed to the Editor.

The latest number of the REGISTER may be ordered with or without other goods at any time, and all letters should be addressed to

